# Supplementary figures and images for: Comparative efficacy of prophylactic anticonvulsant drugs following traumatic brain injury: A systematic review and network meta-analysis of randomized controlled trials
Source: PLoS One. 2022 Mar 31;17(3):e0265932. doi: 10.1371/journal.pone.0265932 (PMC8970384; doi:10.1371/journal.pone.0265932)

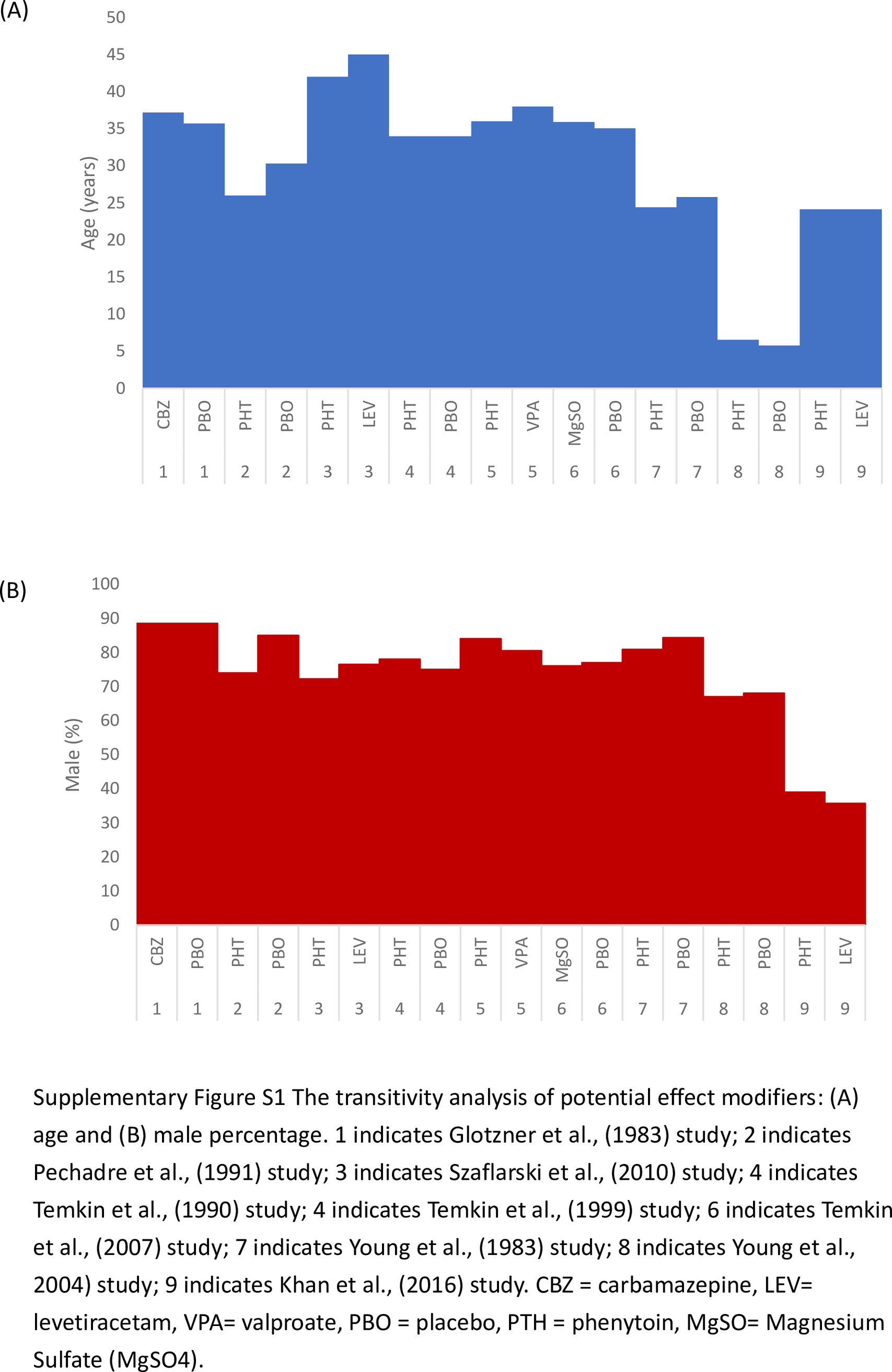

Supplement: S1 Fig — (A) age and (B) male percentage. 1 indicates Glotzner et al., (1983) study; 2 indicates Pechadre et al., (1991) study; 3 indicates Szaflarski et al., (2010) study; 4 indicates Temkin et al., (1990) study; 4 indicates Temkin et al., (1999) study; 6 indicates Temkin et al., (2007) study; 7 indicates Young et al., (1983) study; 8 indicates Young et al., 2004) study; 9 indicates Khan et al., (2016) study. CBZ = carbamazepine, LEV = levetiracetam, VPA = valproate, PBO = placebo, PTH = phenytoin, MgSO = Magnesium Sulfate (MgSO4). (TIF) [file pone.0265932.s001.tif]

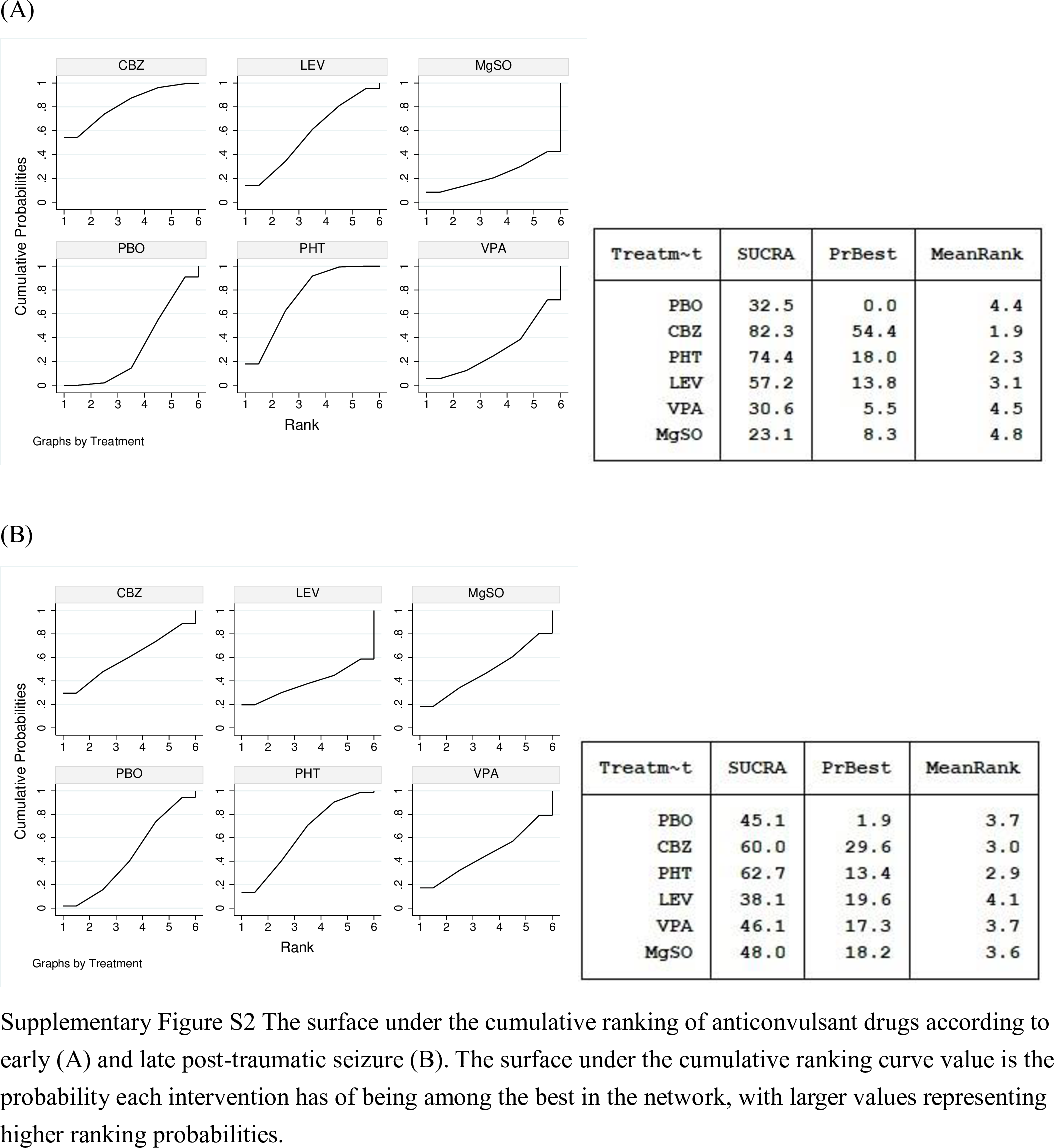

Supplement: S2 Fig — The surface under the cumulative ranking curve value is the probability each intervention has of being among the best in the network, with larger values representing higher ranking probabilities. (TIF) [file pone.0265932.s002.tif]

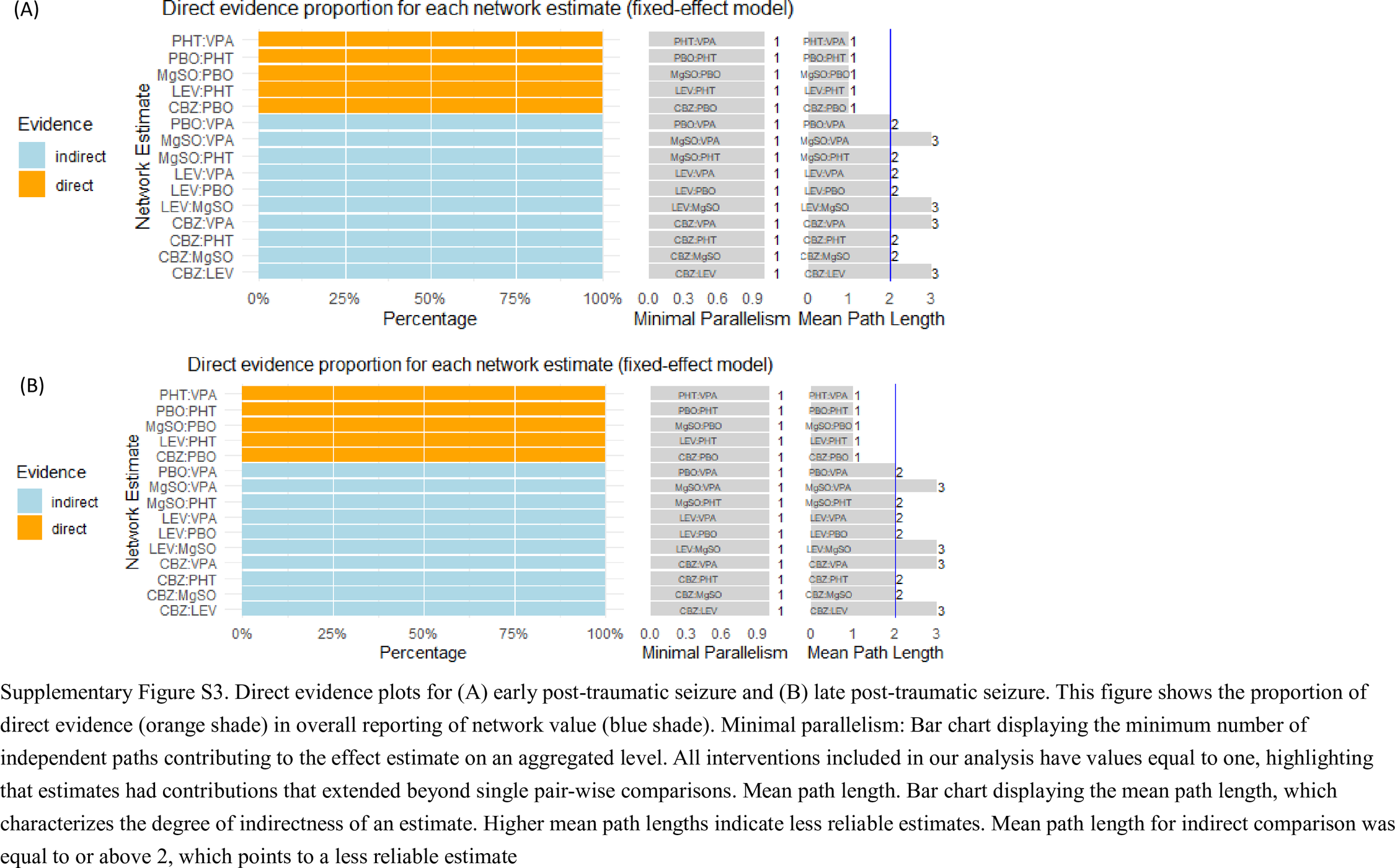

Supplement: S3 Fig — Direct evidence plots for (A) early post-traumatic seizure and (B) late post-traumatic seizure. This figure shows the proportion of direct evidence (orange shade) in overall reporting of network value (blue shade). Minimal parallelism: Bar chart displaying the minimum number of independent paths contributing to the effect estimate on an aggregated level. All interventions included in our analysis have values equal to one, highlighting that estimates had contributions that extended beyond single pair-wise comparisons. Mean path length. Bar chart displaying the mean path length, which characterizes the degree of indirectness of an estimate. Higher mean path lengths indicate less reliable estimates. Mean path length for indirect comparison was equal to or above 2, which points to a less reliable estimate. (TIF) [file pone.0265932.s003.tif]

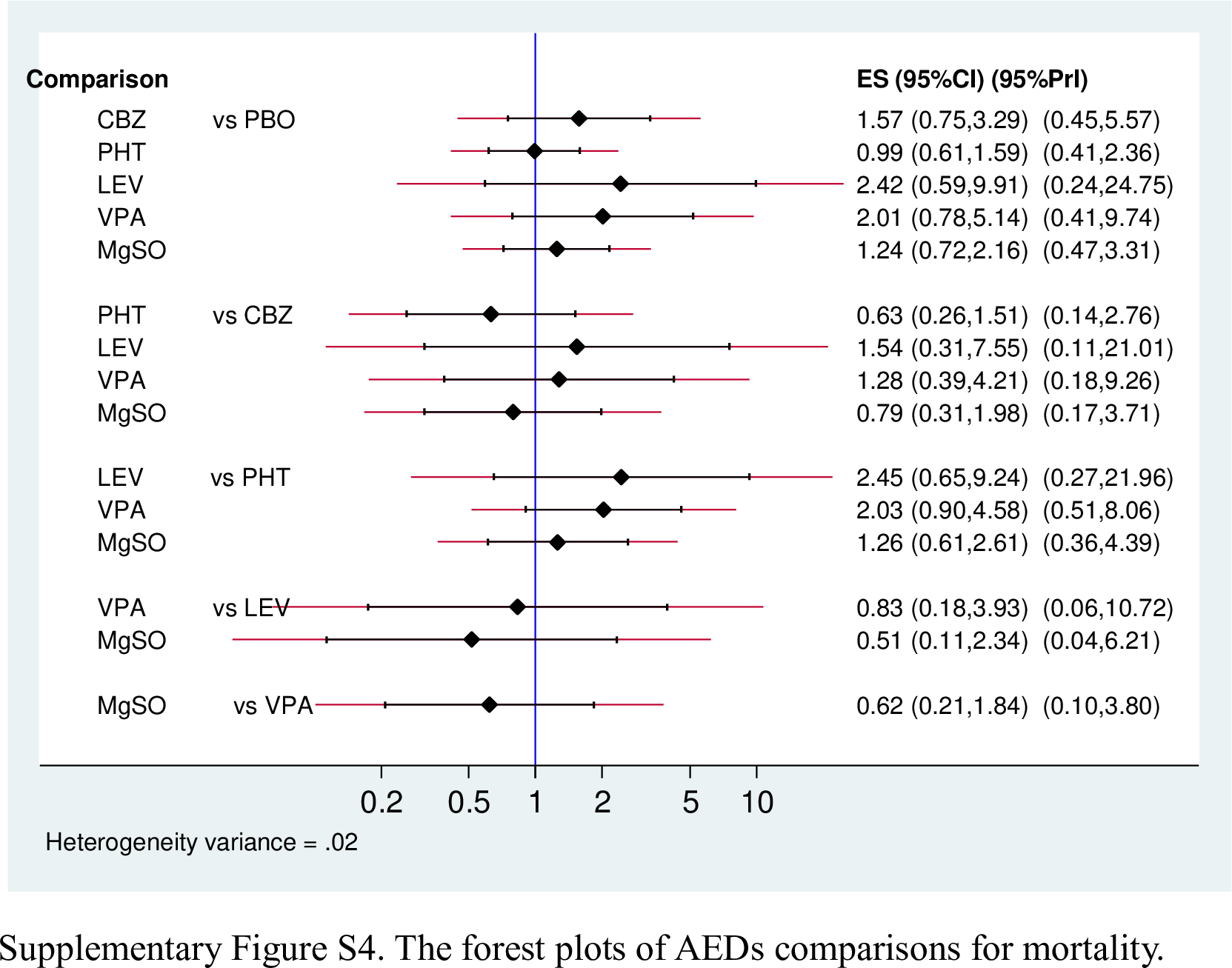

Supplement: S4 Fig — (TIF) [file pone.0265932.s004.tif]
