# Supplementary material for: Comparative efficacy of prophylactic anticonvulsant drugs following traumatic brain injury: A systematic review and network meta-analysis of randomized controlled trials
Source: PLoS One. 2022 Mar 31;17(3):e0265932. doi: 10.1371/journal.pone.0265932 (PMC8970384; doi:10.1371/journal.pone.0265932)
Supplement: S1 Table — (DOCX) [file pone.0265932.s005.docx]

**S1 Table. An example of search strategies**

***Pubmed***

#1 **traumatic brain injur***

#2 **traumatic head injur***

#3 **brain injur***

#4 **head injur***

#5 **craniocerebral trauma***

#6 **craniocerebral injur***

#7 **brain trauma***

#8 **head trauma***

#9 **post-trauma**

#10 **posttrauma**

#11 **post trauma**

#12 **(#1 OR #2 OR #3 OR #4 OR #5 OR #6 OR #7 OR #8 OR #9 OR #10 OR #11)**

#13 **seizure***

#14 **epilep***

#15 **convuls***

#16 **(#13 OR #14 OR #15)**

#17 **anticonvulsant**

#18 **anticonvulsive agent**

#19 **antiepilep***

#20 **anti-epilep***

#21 **antiseizure**

#22 **neuroprotective agents**

#23 **phenytoin**

#24 **phenobarbital**

#25 **carbamazepine**

#26 **magnesium sulfate**

#27 **levetiracetam**

#28 **gabapentin**

#29 **valproate**

#30 **(#17 OR #18 OR #19 OR #20 OR #21 OR #22 OR #23 OR #24 OR #25 OR #26 OR #27 OR #28 OR #29)**

#31 **(#12 AND #16 AND #30)** Filters: **Randomized Controlled Trial**

***Embase***

#1 ‘**traumatic brain injur*’**

#2 ‘**traumatic head injur*’**

#3 ‘**brain injur*’**

#4 ‘**head injur*’**

#5 ‘**craniocerebral trauma*’**

#6 ‘**craniocerebral injur*’**

#7 ‘**brain trauma*’**

#8 ‘**head trauma*’**

#9 **post-trauma**

#10 **posttrauma**

#11 ‘**post trauma’**

#12 **(#1 OR #2 OR #3 OR #4 OR #5 OR #6 OR #7 OR #8 OR #9 OR #10 OR #11)**

#13 **seizure***

#14 **epilep***

#15 **convuls***

#16 **(#13 OR #14 OR #15)**

#17 **anticonvulsant**

#18 ‘**anticonvulsive agent’**

#19 **antiepilep***

#20 **anti-epilep***

#21 **antiseizure**

#22 ‘**neuroprotective agents’**

#23 **phenytoin**

#24 **phenobarbital**

#25 **carbamazepine**

#26 ‘**magnesium sulfate’**

#27 **levetiracetam**

#28 **gabapentin**

#29 **valproate**

#30 **(#17 OR #18 OR #19 OR #20 OR #21 OR #22 OR #23 OR #24 OR #25 OR #26 OR #27 OR #28 OR #29)**

#31 **(#12 AND #16 AND #30)**

#32 **#31 AND** **('randomized controlled trial'/de OR 'randomized controlled trial topic'/de)**

***Cochrane Trials (CENTRAL)***

#1 ‘**traumatic brain injur*’**

#2 ‘**traumatic head injur*’**

#3 ‘**brain injur*’**

#4 ‘**head injur*’**

#5 ‘**craniocerebral trauma*’**

#6 ‘**craniocerebral injur*’**

#7 ‘**brain trauma*’**

#8 ‘**head trauma*’**

#9 **#1 OR #2 OR #3 OR #4 OR #5 OR #6 OR #7 OR #8**

#10 **post-trauma**

#11 **posttrauma**

#12 ‘**post trauma’**

#13 **seizure***

#14 **epilep***

#15 **convuls***

#16 **#10 OR #11 OR #12 OR #13 OR #14 OR #15**

#17 **anticonvulsant**

#18 ‘**anticonvulsive agent’**

#19 **antiepilep***

#20 **anti-epilep***

#21 **antiseizure**

#22 ‘**neuroprotective agents’**

#23 **phenytoin**

#24 **phenobarbital**

#25 **carbamazepine**

#26 ‘**magnesium sulfate’**

#27 **levetiracetam**

#28 **gabapentin**

#29 **valproate**

#30 **#17 OR #18 OR #19 OR #20 OR #21 OR #22 OR #23 OR #24 OR #25 OR #26 OR #27 OR #28 OR #29**

#31 **#9 AND #16 AND #30** in Trials

***Scoups***

(TITLE-ABS-KEY**(“traumatic brain injur*” OR “traumatic head injur*” OR "brain injur*" OR "head injur*" OR "craniocerebral trauma*" OR "craniocerebral injur*" OR "brain trauma*" OR "head trauma*" OR post-trauma OR posttrauma OR "post trauma")**) AND (TITLE-ABS-KEY**(epilep* OR seizure* OR convuls*)**) AND (TITLE-ABS-KEY**(anticonvulsant OR “anticonvulstive agent” OR antiepilep* OR anti-epilep* OR antiseizure OR " neuroprotective agents" OR phenytoin OR phenobarbital OR carbamazepine OR "magnesium sulfate" OR levetiracetam OR gabapentin OR valproate)**) AND (TITLE-ABS-KEY**(randomly OR “clinical trial” OR “controlled trial” OR randomised OR randomized OR placebo*)**) AND (LIMIT-TO (DOCTYPE, “ar”)
