# Supplementary material for: Comparative efficacy of prophylactic anticonvulsant drugs following traumatic brain injury: A systematic review and network meta-analysis of randomized controlled trials
Source: PLoS One. 2022 Mar 31;17(3):e0265932. doi: 10.1371/journal.pone.0265932 (PMC8970384; doi:10.1371/journal.pone.0265932)
Supplement: S2 Table — (DOCX) [file pone.0265932.s006.docx]

| **S2 Table. *Characteristics of Studies*** | | | | | | |
| --- | --- | --- | --- | --- | --- | --- |
| **Author, year** | **County** | **Population** | **Treatment group** | | **Control group** | |
|  |  |  | **Participants and Outcome** | **Interventions** | **Participants and Outcome** | **Interventions** |
| Glotzner, 1983 [25] | DE | 151 participants, > 15 years of age, 88.5 % male, with moderate and severe TBI. | Carbamazepine  -Early seizure: 8/75  -Late seizure: 14/75 | Participants were treated according to serum levels 300-600μg. First dose given immediately after accident (no dosage given) | Placebo  -Early seizure: 22/76  -Late seizure: 20/76 | Details not provided |
| Khan, 2016 [31] | PK | 154 participants, age 7-48 years, mean age of 24.15 +/- 9.56, 74.5% male, with moderate to severe TBI. | Phenytoin  -Early seizure: 4/77 | Participants were starting in a loading dose of 20 mg/kg intravenously over sixty minutes followed by maintenance dose of 5 mg/kg/day in two divided doses. | Levetiracetam  -Early seizure: 7/77 | Participants were starting in a loading dose of 20 mg/kg intravenously over sixty minutes follow by maintenance dose of 10-20 mg/kg/day in two divided doses. |
| Mcqueen, 1983 [30] | UK | 164 participants, ages 5-65 years, 79% male (PHT), 80% male (PBO). | Phenytoin  -Late seizure: 8/84 | Child (5-15 years) 5mg/kg; adults 300mg  *during f/u, adjusted to achieve plasma concentration 40-80 μmol/L. | Placebo  -Late seizure: 7/80 | Matching placebo capsules |
| Pechadre 1991 [26] | FR | 86 participants, ages 5-60 years, 80% males with severe TBI | Phenytoin  -Early seizure: 2/34  -Late seizure: 2/34 | 10 mg/kg by slow IV pump 40 mg/min | Placebo  -Early seizure: 13/52  -late seizure: 22/52 | Matching placebo capsules |
| Szaflarski, 2010 [34] | US | 52 participants, with severe TBI or SAH hemorrhage ages 17-80 years. 77% male (LEV), 72% male (PHT). | Phenytoin  -Early seizure: 3/18  -Late seizure: 0/14 | Loading dose of 20 mg/kg IV, max 2000mg over 60 mins and then maintenance of 5 mg/kg/day rounded to nearest 100 mg, dose ever 12 hrs. *therapeutic dose: 10-20 μg/dL. | Levetiracetam  -Early seizure: 5/34  -Late seizure: 1/20 | Loading dose 20 mg rounded to nearest 250 mg over 60 mins. Maintenance dose of 1000mg, IV every 12 hrs over 15 mins.  *therapeutic dose: up to 1500mg (3000mg/day). Duration of treatment: 1-7 days. |

| **S2 Table. *Characteristics of Studies (continue)*** | | | | | | |
| --- | --- | --- | --- | --- | --- | --- |
| **Author, year** | **County** | **Population** | **Treatment group** | | **Control group** | |
|  |  |  | **Participants and Outcome** | **Interventions** | **Participants and Outcome** | **Interventions** |
| Temkin, 1990 [33] | US | 404 participants, with severe TBI, mean age 34 +/- 18 years. 78% male (PHT), 75% male (PBO). | Phenytoin  -Early seizure: 7/208  -Late seizure: 36/170 | Initial dose 20mg/kg IV within 24 hrs of injury  *therapeutic dose: total 40-80μmol/L, 10-20 mg/L  Dose administration: daily dose varied based on individual serum level: range 200-1200 mg to maintain serum levels. | Placebo  -Early seizure: 26/196  -Late seizure: 26/153 | Given daily |
| Temkin, 1999 [32] | US | 379 participants, with TBI, at least 14 years of age (mean age 36-40 years), 84% meal (PHT). | Phenytoin  -Early seizure: 2/132  -Late seizure: 17/132 | Loading dose IV 20 mg/kg, administered within 24 hrs. maintenance dose 5 mg/kg/day in 2 divided doses.  *therapeutic dose: 40-80μmol/L | Valproate  -Early seizure: 11/247  -Late seizure: 39/221 | Loading dose IV 20 mg/kg. maintenance dose 15 mg/kg/day in 4 divided dose.  *therapeutic dose: 277-693μmol/L. |
| Temkin, 2007 [27] | US | 499 participants, older then 14 years with moderate or severe TBI | *High-dose* MgSO4 (n=59)  *Low-dose* MgSO4 (n=191)  -Early seizure:1/250  -Late seizure:15/249 | High dose 1.2-2.5 mmol/L, initial IV load of 0.425mmol/kg over 15 mins followed by continuous infusion (0.1 mmol/kg/hr) to maintain target range for 5 days.  *therapeutic dose: 1.25-2.5 mmol/L  Low dose 1.0-1.85 mmol/L, initial IV load of 0.3mmol/kg over 15 mins followed by continuous infusion (0.05 mmol/kg/hr) to maintain target range for 5 days.  *therapeutic dose: 1.0-1.85 mmol/L | Placebo 1. (n=59)  Placebo 2. (n=190)  -Early seizure:0/249  -Late seizure:14/249 | saline |

| **S2 Table. *Characteristics of Studies (continue)*** | | | | | | |
| --- | --- | --- | --- | --- | --- | --- |
| **Author, year** | **County** | **Population** | **Treatment group** | | **Control group** | |
|  |  |  | **Participants and Outcome** | **Interventions** | **Participants and Outcome** | **Interventions** |
| Young, 1983(A) [28] | US | 244 participants of all ages with severe TBI | Phenytoin  -Early seizure: 5/136 | initial dose 11 mg/kg at 25 mg/minute plus 13 mg/kg intramuscularly If levels were adequate 8.8 mg/kg administered daily or adjusted as needed. *Therapeutic dose: plasma concentrations 10-20 μg/ml. | Placebo  -Early seizure: 4/108 | identical IV of phenytoin diluent (10% ethanol, propylene glycol 40% and water 50%) or placebo capsule |
| Young, 1983(B) [29] | US | 214 participants of all ages with severe TBI, mean age of 25.2 years | Phenytoin  -Late seizure: 13/105 | initial dose 11 mg/kg at 25 mg/minute plus 13 mg/kg intramuscularly If levels were adequate 8.8 mg/kg administered daily or adjusted as needed. *Therapeutic dose: plasma concentrations 10-20 μg/ml. | Placebo  -Late seizure: 8/74 | identical IV of phenytoin diluent (10% ethanol, propylene glycol 40% and water 50%) or placebo capsule |
| Young, 2004 [20] | US | 103 participants, ages <10 years with moderate and severe TBI, 68% male | Phenytoin  -Early seizure: 3/46 | initial IV dose 18 mg/kg over 20 minutes, maintenance 2 mg/kg every 8 hours for 48 hours (5 doses). | Placebo  -Early seizure:3/56 | diluent alone |
| *Participants and Outcome: number of seizure attack/all participants of treatment or control group | | | | | | |
