# Supplementary material for: Comparative efficacy of prophylactic anticonvulsant drugs following traumatic brain injury: A systematic review and network meta-analysis of randomized controlled trials
Source: PLoS One. 2022 Mar 31;17(3):e0265932. doi: 10.1371/journal.pone.0265932 (PMC8970384; doi:10.1371/journal.pone.0265932)
Supplement: S3 Table — (DOCX) [file pone.0265932.s007.docx]

S3 Table. Pairwise meta-analytic results for early and late posttraumatic seizures

| Treatment | Control | No. of studies | OR | (95% CI) | Heterogeneity | | | |
| --- | --- | --- | --- | --- | --- | --- | --- | --- |
|  |  |  |  |  | I^2^ | Q | P | Tau^2^ |
| Early |  |  |  |  |  |  |  | 0 |
| CBZ | PBO | 1 | 0.29 | (0.12 to 0.71) |  |  |  | 0 |
| PHT | PBO | 4 | 0.44 | (0.17 to 1.11) | 50.6% | 6.08 | 0.11 | 0.45 |
| LEV | PHT | 2 | 1.35 | (0.50 to 3.63) | 0% | 0.53 | 0.47 | 0 |
| VPA | PHT | 1 | 3.03 | (0.66 to 13.88) |  |  |  | 0 |
| MgSO | PBO | 1 | 3.00 | (0.12 to 74.00) |  |  |  | 0 |
| Overall (CBZ+PHT+MgSO) | PBO | 6 | 0.42 | (0.21 to 0.82) | 37.5% | 7.98 | 0.16 | 0.25 |
| Late |  |  |  |  |  |  |  | 0 |
| CBZ | PBO | 1 | 0.64 | (0.30 to 1.39) |  |  |  | 0 |
| PHT | PBO | 4 | 0.75 | (0.30 to 1.86) | 72.7% | 10.97 | 0.01 | 0.60 |
| LEV | PHT | 1 | 2.23 | (0.08 to 58.81) |  |  |  | 0 |
| VPA | PHT | 1 | 1.45 | (0.78 to 2.68) |  |  |  | 0 |
| MgSO | PBO | 1 | 1.08 | (0.51 to 2.28) |  |  |  | 0 |
| Overall (CBZ+PHT+MgSO) | PBO | 6 | 0.82 | (0.47 to 1.43) | 59.7% | 12.15 | 0.04 | 0.27 |

OR = odds ratio, CI= confidence interval, PBO = placebo, CBZ = carbamazepine, PTH = phenytoin, LEV = levetiracetam, VPA = valproate, MgSO= Magnesium Sulfate (MgSO_4_), T = treatment, C = control.
