# Supplementary material for: Comparative efficacy of prophylactic anticonvulsant drugs following traumatic brain injury: A systematic review and network meta-analysis of randomized controlled trials
Source: PLoS One. 2022 Mar 31;17(3):e0265932. doi: 10.1371/journal.pone.0265932 (PMC8970384; doi:10.1371/journal.pone.0265932)
Supplement: S4 Table — (DOCX) [file pone.0265932.s008.docx]

S4 Table. League tables for comparing anticonvulsant drugs for early and late post-traumatic seizures.

| **Early PTS (n = 9)** | | | | | |
| --- | --- | --- | --- | --- | --- |
| PBO | 0.29 (0.07,1.18) | 0.43 (0.18,1.01) | 0.56 (0.12,2.55) | 1.29 (0.17,10.07) | 3.00 (0.10,88.12) |
| 0.29 (0.12, 0.71) | CBZ | 1.45 (0.28,7.49) | 1.91 (0.25,14.96) | 4.41 (0.37,52.63) | 10.24 (0.26,395.82) |
| 0.44 (0.17, 1.11) |  | PHT | 1.32 (0.38,4.60) | 3.03 (0.47,19.50) | 7.04 (0.21,230.62) |
|  |  | 1.35 (0.50, 3.63) | LEV | 2.30 (0.24,21.69) | 5.35 (0.13,217.05) |
|  |  | 3.03 (0.66, 13.88) |  | VPA | 2.32 (0.04,121.26) |
| 3.00 (0.12, 74.00) |  |  |  |  | MgSO |
| **Late PTS (n=8)** | | | | | |
| PBO | 0.64 (0.08,5.28) | 0.71 (0.23,2.20) | 1.58 (0.03,84.42) | 1.03 (0.10,10.72) | 1.08 (0.13,8.78) |
| 0.64 (0.30,1.39) | CBZ | 1.10 (0.10,12.05) | 2.46 (0.03,221.72) | 1.60 (0.07,37.39) | 1.67 (0.09,32.77) |
| 0.75 (0.30, 1.86) |  | PHT | 2.23 (0.05,101.13) | 1.45 (0.19,11.31) | 1.52 (0.14,16.50) |
|  |  | 2.23 (0.08,58.81) | LEV | 0.65 (0.01,49.46) | 0.68 (0.01,61.21) |
|  |  | 1.45 (0.78, 2.68) |  | VPA | 1.05 (0.05,24.40) |
| 1.08 (0.51, 2.28) |  |  |  |  | MgSO |
| **Sensitivity analysis for early PTS (n = 8)** | | | | | |
| PBO | 0.29 (0.09,1.01) | **0.33 (0.14,0.78) ^*^** | 0.44 (0.10,1.86) | 1.00 (0.14,7.04) | 3.00 (0.11,82.84) |
| 0.29 (0.12, 0.71) | CBZ | 1.13 (0.25,5.09) | 1.50 (0.22,9.99) | 3.42 (0.34,34.34) | 10.24 (0.30,352.74) |
| 0.34 (0.13, 0.89) |  | PHT | 1.33 (0.41,4.25) | 3.03 (0.53,17.38) | 9.06 (0.29,279.29) |
|  |  | 1.35 (0.50, 3.63) | LEV | 2.28 (0.28,18.65) | 6.83 (0.18,254.47) |
|  |  | 3.03 (0.66, 13.88) |  | VPA | 2.99 (0.06,140.22) |
| 3.00 (0.12, 74.00) |  |  |  |  | MgSO |

*indicates p < 0.05. left bottom column: results of direct head-to-head comparison among treatments; right up column: results of combining direct and indirect comparisons of treatments. A value less than 1 indicates that patients who underwent the treatment in the corresponding cell in the right-hand column had a less risk of developing post-traumatic seizures (PTS) than patients who underwent the treatment in the corresponding cell in the top row, and a value greater than 1 indicates the opposite. Abbreviations: Abbreviations: PBO = placebo, CBZ = carbamazepine, PTH = phenytoin, LEV = levetiracetam, VPA = valproate, MgSO= Magnesium Sulfate (MgSO_4_).
