# Supplementary material for: Comparative efficacy of prophylactic anticonvulsant drugs following traumatic brain injury: A systematic review and network meta-analysis of randomized controlled trials
Source: PLoS One. 2022 Mar 31;17(3):e0265932. doi: 10.1371/journal.pone.0265932 (PMC8970384; doi:10.1371/journal.pone.0265932)
Supplement: S5 Table — (DOCX) [file pone.0265932.s009.docx]

S5 Table. Risk of methodological bias score of the studies (n=11)

| First Authors, published year | Randomization  process | Deviations from intended | Missing outcome data | Measurement of the outcome | Selection of the reported result | Overall bias |
| --- | --- | --- | --- | --- | --- | --- |
| Glotzner, 1983 [22] | S | H | L | S | S | H |
| Khan, 2016 [29] | L | L | L | L | L | L |
| Mcqueen, 1983 [28] | L | L | S | S | L | S |
| Pechadre 1991 [23] | S | H | L | S | H | H |
| Szaflarski, 2010 [32] | S | L | L | S | L | S |
| Temkin, 1990 [31] | S | L | L | S | L | S |
| Temkin, 1999 [30] | S | L | S | S | L | S |
| Temkin, 2007 [24] | L | L | L | S | L | S |
| Young, 1983(A) [25] | S | S | L | L | H | H |
| Young, 1983(B) [26] | S | S | L | L | H | H |
| Young, 2004 [27] | S | L | L | S | L | S |

L= low risk; H=high risk; S= some concerns.
