# Supplementary material for: Comparative efficacy of prophylactic anticonvulsant drugs following traumatic brain injury: A systematic review and network meta-analysis of randomized controlled trials
Source: PLoS One. 2022 Mar 31;17(3):e0265932. doi: 10.1371/journal.pone.0265932 (PMC8970384; doi:10.1371/journal.pone.0265932)
Supplement: S6 Table — (DOCX) [file pone.0265932.s010.docx]

**S6A Table. CINeMA summary tables for early PTS**

|  | Comparison | Within-study bias | Reporting bias | Indirectness | Imprecision | Heterogeneity | Incoherence | Confidence rating | Downgrade reasons |
| --- | --- | --- | --- | --- | --- | --- | --- | --- | --- |
| Mixed Evidence | CBZ:PBO | Major concerns | Undetected | No concerns | Major concerns | No concerns | Major concerns | Very low | Within-study bias, Imprecision, Incoherence |
|  | LEV:PHT | No concerns | Undetected | Some concerns | Major concerns | No concerns | Major concerns | Low | Indirectness, Imprecision, Incoherence |
|  | MgSO:PBO | Some concerns | Undetected | No concerns | Major concerns | No concerns | Major concerns | Low | Within-study bias, Imprecision, Incoherence |
|  | PBO:PHT | Major concerns | Undetected | No concerns | No concerns | Major concerns | Major concerns | Very low | Within-study bias, Heterogeneity, incoherence |
|  | PHT:VPA | Some concerns | Undetected | No concerns | Major concerns | No concerns | Major concerns | Low | Within-study bias, Imprecision, Incoherence |
|  | CBZ:LEV | Some concerns | Undetected | No concerns | Major concerns | No concerns | Major concerns | Low | Within-study bias, Imprecision, Incoherence |
|  | CBZ:MgSO | Major concerns | Undetected | No concerns | Major concerns | No concerns | Major concerns | Very low | Within-study bias, Imprecision, Incoherence |
| Indirect Evidence | CBZ:PHT | Major concerns | Undetected | No concerns | Major concerns | No concerns | Major concerns | Very low | Within-study bias, Imprecision, Incoherence |
|  | CBZ:VPA | Major concerns | Undetected | No concerns | Major concerns | No concerns | Major concerns | Very low | Within-study bias, Imprecision, Incoherence |
|  | LEV:MgSO | Some concerns | Undetected | No concerns | Major concerns | No concerns | Major concerns | Low | Within-study bias, Imprecision, Incoherence |
|  | LEV:PBO | Some concerns | Undetected | No concerns | Major concerns | No concerns | Major concerns | Low | Within-study bias, Imprecision, Incoherence |
|  | LEV:VPA | Some concerns | Undetected | No concerns | Major concerns | No concerns | Major concerns | Low | Within-study bias, Imprecision, Incoherence |
|  | MgSO:PHT | Some concerns | Undetected | No concerns | Major concerns | No concerns | Major concerns | Low | Within-study bias, Imprecision, Incoherence |
|  | MgSO:VPA | Some concerns | Undetected | No concerns | Major concerns | No concerns | Major concerns | Low | Within-study bias, Imprecision, Incoherence |
|  | PBO:VPA | Some concerns | Undetected | No concerns | Major concerns | No concerns | Major concerns | Low | Within-study bias, Imprecision, Incoherence |

**S6B Table CINeMA summary tables for late PTS**

|  | Comparison | Within-study bias | Reporting bias | Indirectness | Imprecision | Heterogeneity | Incoherence | Confidence rating | Downgrading Reasons |
| --- | --- | --- | --- | --- | --- | --- | --- | --- | --- |
| Mixed Evidence | CBZ:PBO | Major concerns | Undetected | No concerns | Major concerns | No concerns | Major concerns | Very low | Within-study bias, Imprecision, Incoherence |
|  | LEV:PHT | Some concerns | Undetected | No concerns | Major concerns | No concerns | Major concerns | Low | Within-study bias, Imprecision, Incoherence |
|  | MgSO:PBO | Some concerns | Undetected | No concerns | Major concerns | No concerns | Major concerns | Low | Within-study bias, Imprecision, Incoherence |
|  | PBO:PHT | Some concerns | Undetected | No concerns | Major concerns | No concerns | Major concerns | Low | Within-study bias, Imprecision, Incoherence |
|  | PHT:VPA | Some concerns | Undetected | No concerns | Major concerns | No concerns | Major concerns | Low | Within-study bias, Imprecision, Incoherence |
| Indirect Evidence | CBZ:LEV | Some concerns | Undetected | No concerns | Major concerns | No concerns | Major concerns | Low | Within-study bias, Imprecision, Incoherence |
|  | CBZ:MgSO | Some concerns | Undetected | No concerns | Major concerns | No concerns | Major concerns | Low | Within-study bias, Imprecision, Incoherence |
|  | CBZ:PHT | Major concerns | Undetected | No concerns | Major concerns | No concerns | Major concerns | Very low | Within-study bias, Imprecision, Incoherence |
|  | CBZ:VPA | Some concerns | Undetected | No concerns | Major concerns | No concerns | Major concerns | Low | Within-study bias, Imprecision, Incoherence |
|  | LEV:MgSO | Some concerns | Undetected | No concerns | Major concerns | No concerns | Major concerns | Low | Within-study bias, Imprecision, Incoherence |
|  | LEV:PBO | Some concerns | Undetected | No concerns | Major concerns | No concerns | Major concerns | Low | Within-study bias, Imprecision, Incoherence |
|  | LEV:VPA | Some concerns | Undetected | No concerns | Major concerns | No concerns | Major concerns | Low | Within-study bias, Imprecision, Incoherence |
|  | MgSO:PHT | Some concerns | Undetected | No concerns | Major concerns | No concerns | Major concerns | Low | Within-study bias, Imprecision, Incoherence |
|  | MgSO:VPA | Some concerns | Undetected | No concerns | Major concerns | No concerns | Major concerns | Low | Within-study bias, Imprecision, Incoherence |
|  | PBO:VPA | Some concerns | Undetected | No concerns | Major concerns | No concerns | Major concerns | Low | Within-study bias, Imprecision, Incoherence |
