# Supplementary material for: Comparative efficacy of prophylactic anticonvulsant drugs following traumatic brain injury: A systematic review and network meta-analysis of randomized controlled trials
Source: PLoS One. 2022 Mar 31;17(3):e0265932. doi: 10.1371/journal.pone.0265932 (PMC8970384; doi:10.1371/journal.pone.0265932)
Supplement: Attachment — Submitted filename: response to reviewers comment-0905 HYC.docx [file pone.0265932.s011.docx]

Reviewer #1: This manuscript is a network meta-analysis (NMA) of randomised controlled trials performed to evaluate the effect of prophylactic anticonvulsant therapy in patients with traumatic brain injury. The topic is of interest and certainly an area of equipoise in current practice. Current guidelines suggest the use of empiric anticonvulsant therapy in patients presenting with severe traumatic brain injury for up to one week, but practice has been demonstrated in surveys to vary widely among neurosurgeons. The authors’ analysis of the twelve included studies suggests no benefit to any individual antiepileptic drug. Overall, this is a useful and important topic with interesting implications that might be somewhat answered using an NMA. However, there are methodological flaws which should be addressed before publication can be considered.

Response: Thank you for your thoughtful review. We have carefully studied your comments and suggestions and revised our paper accordingly. The following are our point-by-point responses to your specific comments. We hope our revisions are acceptable and our responses adequately address the comments. Thank you for your consideration.

Statistical analysis: Lacking in detail, see comments below. However, the treatment ranking described by the authors is reproducible from the data provided.

Response: Thank you for the comment. We have revised the statistical analysis according to your suggestion.

English language/grammar: Requires further proof-reading.
Response: Thank you for the comment. We have had the manuscript reviewed by a native English speaker with scientific expertise to ensure proper English grammar and usage.

Major comments:
1. Firstly, the authors should consider whether an NMA is appropriate. I note that, of the 5 drugs studied for the prevention of early seizures for example, 4 were compared in only a single study arm which limits power somewhat. An NMA, if performed, should be very carefully evaluated for integrity in such an instance.
Response: Thank you for the thoughtful comment. We agree with your concerns that the NMA may have caused reduced statistical power, thus threatening the internal validity of our findings. However, compared with traditional pairwise meta-analysis, the strength of the NMA lies in comparing different AEDs simultaneously. Even so, we have emphasized this concern in the section of Discussion and as one of the study limitations as follows: *It is worth noting that the two pairwise meta-analyses [8, 9] that pooled relevant studies together may have substantially increased statistical power by doing so. By contrast, NMA, which our study adopted, may have led to few treatment comparison studies, causing reduced statistical power [33]. Our study results should therefore be interpreted with caution, with future studies required to verify our findings (Discussion section); Several limitations should be considered when interpreting our data. First,…Second, only a few studies were included for single study arm comparison, thus limiting the statistical power.…(Limitations)*

2. How were early and late seizures defined? This is important for interpretation and also ensuring transitivity in the NMA and as such should be clearly stated in the methods section.
Response: We agree with your comment that clear definitions of early and late seizures are critical for interpretation and ensuring transitivity in the NMA. We have added the definition in the Material and Methods section as follows (P.4): *“Early PTS” refers to the occurrence of seizures within 1 week of head injury, and “late PTS” refers to seizures occurring 1 week or later after head trauma.*

3. Transitivity is perhaps the most crucial assumption of the NMA and should be defined and assessed by the authors. This should be explained in the methods section.

Response: We have defined and assessed transitivity, the most crucial assumption of the NMA in the methods section as follow (p 7): *Transitivity, which refers to an absence of systematic differences between the available comparisons other than the treatments being compared, is a crucial assumption for an NMA [20]. Transitivity was qualitatively assessed by the authors; if a substantial variation between comparisons on effect modifiers was identified, intransitivity was suggested [20].* We have also qualitatively described the details of the included studies to confirm the issue of transitivity. We observed that age variations may cause intransitivity; hence, sensitivity analysis was further performed. All details can be found in the Results section (page 8 to 9).

4. The exact search strategy used should be provided (as supplementary) for each database in such a way that it is fully reproducible. This should ideally comply with the PRISMA-P statement.

Response: Thank you for the comments. We have provided the exact search strategy (as Supplementary table 1) for each database, which ideally complies with the PRISMA-P statement.

5. The authors report to have assessed risk of bias in individual studies using the Cochrane Handbook, providing a reference from 2011. Risk of bias in randomised trials is more appropriately assessed using an updated, validated tool such as the RoB 2.0.

Response: Thank you for the comments. We have reassessed the study quality using RoB 2.0. All changes are marked in red in both the Material and Methods and the Results sections.

6. The authors report to have performed pairwise meta-analysis in addition to NMA for each pairing, but do not appear to have reported the results. Pairwise analyses should be reported separately from and clearly delineated from the network estimates derived from the NMA, perhaps in league table form.

Response: Thank you for the comments. We did perform the pairwise meta-analysis before the NMA. We have added the pairwise analyses in a league table format.

7. The authors mention that random effects regression was applied to the pairwise analyses. Was a random effects model also used for the network estimates? This should be clarified.

Response: Thank you for the comments. This was a typo. We have deleted the sentence describing random effects regression and noted the random-effects model in the Data analysis section as follows: *The network meta-analysis was conducted under a frequentist framework with a random-effects model. (Page 7)*

8. The authors mention the calculation of a SUCRA treatment ranking and SUCRA ranking positions are alluded to in the manuscript but the treatment ranking for each category studied is not overtly provided. These rankings should be included in tabular form for each analysis with the exact SUCRA scores provided. A cumulative ranking plot may also be of benefit to illustrate.

Response: Thank you for your suggestions. We have added SUCRA plots with exact SCURA scores for each outcome in Supplementary Figure S1.

9. SUCRA is a useful metric in the NMA but NMA is a relatively novel concept with which many readers will be unfamiliar and its calculation, implications and interpretations should therefore be explained in the methods.

Response: Thank you for your suggestions. We have added the implications and interpretations of the SUCRA score in the section of Data Analysis as follows: *We ranked probabilities for each treatment, and the surface under the cumulative ranking (SUCRA) curve was employed to facilitate the evaluation of relative treatment efficacy [16, 18]. A higher SUCRA score (range, 0%–100%) indicated a greater likelihood of therapy in the top rank or one of the top ranks [19].*

10. On page 8, the authors mention that Egger’s p < 0.05 indicated no significant publication bias. This is incorrect - p < 0.05 indicates significant asymmetry and suggests the presence of publication bias.

Response: It was a typo. We have provided the exact *p*-value indicative of no significant publication bias.

11. The full results of the NMA including effect size estimates with associated confidence intervals and SUCRA scores should be reported for each analysis separately in the text and in the abstract.

Response: Thank you for your suggestion. We have added the effect size estimates with associated CIs and SUCRA scores for each outcome separately in the main text and abstract.

12. The study by Manaka (1992) refers to a control group consisting of “usual care”, but the authors mention that some patients received anticonvulsants. There is no node relating to “usual care” in the network plots – was this group considered equivalent to placebo control? If so, this should be overtly stated as this may violate the transitivity assumption of the NMA.

Response: Thank you for your suggestion. After reviewing the article, we found that usual care arm is not equivalent to placebo, causing the study (Manaka [1992]) could bot connect with NMA map. We therefore removed it from final analysis.

13. The authors mention performing meta-regression in the methods (page 6) but no further detail was provided. If this was performed, please provide details of the statistical methods and software used for this in the methods along with results including regression coefficients, residual heterogeneity and significance levels in the results section.

Response: Thank you for your suggestions. We have deleted this sentence because we did not perform meta-regression due to the inclusion of small-size number of studies.

14. The exploration of inconsistency is mentioned. Where available, the results of the node-splitting analysis performed should be provided for each comparison in the network. Heterogeneity within designs and between designs should be clearly quantitatively reported for each analysis.

Response: Thank you for your suggestions. Because the network plots did not form a closed loop, the pairwise comparisons were based on direct or indirect evidence. That is, inconsistency was not evaluated for the networks. We have mentioned this point in the Network Plots section.

15. The forest plots are currently rather illegible. The axes should be decompressed as the numbers are currently unreadable and the treatment identifiers (A, B, etc) should probably just be substituted for the full treatment names or three-letter identifiers for each treatment eg. PHT for phenytoin. In addition, the legend is absent from figure 5.

Response: Thank you for the comments. To enhance the readability of our data, we have replaced the forest plots with league tables to present the results of treatment comparisons.

16. The authors conclude by stating that clinicians should incorporating their findings, that no anticonvulsant drug significantly decreases the likelihood of experiencing post-traumatic epilepsy, into clinical practice. I find this excessively assured. The authors did not attempt to pool all anticonvulsants into a pairwise anticonvulsants versus placebo meta-analysis, and a Cochrane review on the topic has demonstrated a lower likelihood of seizures when all anticonvulsants are considered together. It is possible, and even likely, that studies of individual treatments are underpowered to demonstrate an effect but when pooled demonstrate that prophylactic anticonvulsant therapy may in fact decrease the likelihood of post-traumatic seizures. The authors refer to this on page 9-10, stating that the study has better understanding of the individual treatments as a result, but consideration should also be given to the increased statistical power achieved by pooling the individual therapies.

Response: We agree with your comment; hence, we have attributed increased statistical power to the pairwise meta-analyses that pooled the individual therapies, and stated that our network meta-analysis involved only a few studies for each comparison in the Discussion section as follows: *It is worth noting that the two pairwise meta-analyses [8, 9] that pooled relevant studies may have substantially increased statistical power by doing so. By contrast, NMA, which our study adopted, may have led to few treatment comparison studies, causing reduced statistical power [33]. Our study results therefore should be interpreted with caution, with future studies required to verify our findings.*

Minor comments:
1. The authors should be commended for reporting raw data in Table 1, but this should be reported separately also as supplementary, ideally in spreadsheet format to allow easier reproduction of the results.

Response: Thank you for the suggestion. We have provided the raw data in spreadsheet format as a supplementary Table S3.

2. I find the choice of skin rash as the only adverse effect to be investigated unconvincing. While it may be the most common, its clinical relevance in patients with severe traumatic brain injury is questionable and perhaps more serious adverse effects should also be considered despite being less common? If this is retained, it should be defined – skin rash associated with anticonvulsants ranges from mild cutaneous irritation to life-threatening dermatological syndromes.

Response: Thank you for the suggestion. After rechecking the included studies, original authors did not define skin rash in the papers; therefore, we could not identify the effects of skin rash on TBI survivors. We have deleted all relevant context regarding the adverse effects of AEDs on skin rash from the manuscript.

3. There are some references missing in the discussion wherein authors are mentioned but no superscript reference is provided eg. by Zafar et al. on page 9.

Response: We have added citation numbers for the missing references.

4. The authors should consider evaluating mean path length (1) given that many treatments are represented by a single study arm.
Response: We have added the average path length to depict the numbers of treatments in a single study arm (Figure 2).

Reviewer #2: In this interesting systematic review the authors aimed was to systematically compare the effects and safety of prophylactic anticonvulsant drug use in patient with traumatic brain injury. Network meta-analysis using multivariate random effects and frequentist framework was applied. A total of 12 randomized controlled trials involving 1,431 participants were included. The authors underline that this study suggests no beneficial effect of anticonvulsant drug on early and late post-traumatic seizure, which does not support the recommendations of the clinical guideline of using Phenytoin as the first-line therapy in treatment of early post-traumatic seizure. The manuscript is well written and it could have a relevant impact on the readership. However, there are few minor points that need to be clarified.
Response: Thank you for your encouraging words. We have revised our manuscript according to your suggestions, and the following are our point-by-point responses to your specific comments. We hope that our revisions are acceptable and that our responses adequately address your comments. Thank you for your consideration.

Specific comments:
- The sentence reported in the abstract “In comparison with placebo, phenytoin, valproate, levetiracetam, carbamazepine, and MgSO4 could significantly reduce the early post-traumatic seizure; and phenytoin, valproate, levetiracetam, carbamazepine, MgSO4, and phenobarbital were not significantly superior to placebo.” is not clear; please reformulate it.

Response: We have revised the abstract as follows: *We found that phenytoin (odd ratios [ORs] = 0.43 and 0.71; 95% confidence intervals [CIs] = 0.18–1.01 and 0.23–2.20), levetiracetam (ORs = 0.56 and 1.58; 95% CIs = 0.12–2.55 and 0.03–84.42), and carbamazepine (OR= 0.29 and 0.64; 95% CIs = 0.07–1.18 and 0.08–5.28) were more likely to reduce early and late PTS compared with placebo; however, the treatment effects were not significant.*

- page 9: please change “seizure” with “seizures”

Response: Because the abbreviation of posttraumatic seizures (i.e., PTS) was defined upon first usage, we have replaced “posttraumatic seizures” with “PTS” throughout the manuscript.

- Please check the different values included in the flow diagram of the study selection process.

Response: We have rechecked and updated the values included in the flow diagram. All changes are marked in red.
