# Supplementary material for: Comparative efficacy of prophylactic anticonvulsant drugs following traumatic brain injury: A systematic review and network meta-analysis of randomized controlled trials
Source: PLoS One. 2022 Mar 31;17(3):e0265932. doi: 10.1371/journal.pone.0265932 (PMC8970384; doi:10.1371/journal.pone.0265932)
Supplement: Attachment — Submitted filename: response to reviewers comment-0121.docx [file pone.0265932.s012.docx]

Reviewer #1: The authors should be commended for a substantial review, which has significantly improved the manuscript. The plots are significantly improved and more legible, and the writing has significantly improved. I have the following comments:
Response: Thank you for your valuable comments that have helped improve our manuscript. We have carefully studied your comments and suggestions and revised our paper accordingly. The following are our point-by-point responses to your specific comments. We hope that our revisions are acceptable and that our responses adequately address the comments.

Major comments:
1. The authors now provide a definition of transitivity as suggested. However, they mention that transitivity was assessed qualitatively only (methods, page 7-8). They do not appear to have defined specific potential effect modifiers, or collected arm-level data on these modifiers. This insufficient to assess the plausibility of transitivity, especially in the context of a star-shaped network, which limits the assessment of its statistical manifestation (inconsistency). Ideally, the authors should define potential effect modifiers and compare their distribution across treatment nodes in the network.
Response: We have added the results of the transitivity assessment to the Results section (page 7). Because not all included studies reported effect modifiers, we only extracted age and male percentage to evaluate transitivity in the NMA. We have stated the results for transitivity as follows: *The transitivity of potential effect modifiers (age and male percentage) is presented in Supplementary Figure S1. Their distributions were not significantly different across treatments, except for Young’s (2004) study for young age and Khan’s (2016) study for low male percentage, indicating potential threats to the transitivity assumption.* In addition, we have added S1 Fig. to present the transitivity assessment results.

2. On this note, the network heterogeneity, quantified by tau^2, should also be reported. I am aware that, for the late seizure analysis in particular, this will be a skewed estimate given that most treatments are comprised of only a single study. However, some effort to quantify the heterogeneity is needed.
Response: We have added tau-squared and I^2^ statistics to the Results section to explain network heterogeneity. All changes are indicated in red font.

3. The authors now report to have included the odds ratios, including direct pairwise estimates, in league table format as Table 2. This does not appear to have been provided.
Response: In addition to league table (S4 Table), we have added details regarding direct pairwise estimates to the Results section and to S3 Table.

4. The authors have now removed the skin rash adverse event from the NMA assessment, which seems appropriate. However, there is now no assessment of adverse events or mortality, which is a significant limitation for any clinician reading, because this informs treatment choice as much as, or even more than efficacy. Ideally, the authors should record data on the number of adverse events recorded and the mortality rate between the various treatments evaluated. This may not be appropriate for network meta-analysis, but should at least be recorded, displayed and discussed.
Response: In addition to skin rash, adverse events and mortality were documented in the included studies. We have added details regarding the occurrence of adverse events and mortality to the Results section (subheading: Adverse events) and addressed associated topics in the Discussion section (page 12). All changes are indicated in red font.

5. The authors claim to now have provided the mean path length for comparisons. What is provided in the plots is in fact the number of studies forming the comparison, as far as I can see. The mean path length I refer to is as described by Konig et al. (2013), below.
Response: We have added the following description of mean path length to the Data Analysis section: *We evaluated the proportion of direct and indirect evidence contributing to each comparison by using the direct evidence plot, which enables us to estimate the minimum number of independent paths in the network contributing to the effect estimate at an aggregated level. According to König et al., minimal parallelism and mean path length are used to estimate the degree of indirectness in the reported pooled outcome, with lower values for minimal parallelism and mean path length > 2 indicating that results for a specific comparison should be interpreted with caution because additional similarity assumptions must be made when each direct comparison is serially combined*.

We have added the following results to the Results section: *Of the 15 unique treatment comparisons, only 5 contained entire direct evidence (proportion = 100%), whereas the remaining 10 were based entirely on indirect evidence (S3 Fig). For all estimates, the minimum number of independent paths contributing to the effect size estimate on an aggregated level (minimal parallelism) was 1, implying less robust estimates. We observed mean path lengths >2 in 4 of the 15 estimates; this implies that the estimate in question should be interpreted with caution*.

All changes are indicated in red font.

6. It is not emphasised enough that anticonvulsants as a whole are superior to placebo for the prevention of early seizures. This has been strongly shown by the Cochrane review. In addition, if studies comparing an antiepileptic drug to placebo in the authors' provided data are meta-analysed (any AED vs. placebo) the summary result from a random effects model is OR 0.42, 95%CI 0.42, p = 0.01, I^2 = 37.5%, tau^2 = 0.25. To me, this is a central point that should be better emphasised by the authors. When this meta-analysis is also performed, the findings suggest that anticonvulsants as a whole are superior to placebo, but the various drugs appear to have equivalent efficacy.
Response: We have described the results of the pairwise meta-analyses in the Results section. We have also emphasized that the AEDs were superior to the placebo for early but not late PTS in the first paragraph of the Discussion section. All changes are indicated in red font.

7. In my opinion, the conclusion that PHT may be a beneficial treatment, without reference to the aggregate of anticonvulsant drugs, is rather unsupported as a result. This is especially given the case that phenytoin did not rank highest in the NMA, did not have superior efficacy to any of the individual drugs in the point estimates and was only superior to placebo in a seemingly arbitrary sensitivity analysis.

Response: We agree with your comments and have rewritten the conclusion as follows: *Although the pairwise MA suggests that AEDs are superior to placebo in preventing early PTS, the NMA identified no AED as superior to another in preventing early or late PTS. Our results do not support the treatment guidelines regarding the prophylactic use of phenytoin in preventing early PTS.* All changes are indicated in red font.

8. In line with recent updates to the PRISMA guidelines, the authors confidence in their findings should be discussed. In network meta-analysis, the CINeMA tool provides a framework and associated web application for this (referenced below).

Response: We used CINeMA to evaluate the certainty of evidence produced by the synthesis for outcomes of early and late PTS. We have described the details regarding CINeMA in the Data Analysis section (page 7) and reported the results in the Results section (page 10). All changes are indicated in red font.

Reviewer #2: The Authors have edited the manuscript as suggested, furthermore I have no further comments to add.

Response: Thank you for your thoughtful review.
