# Supplementary material for: Comparative efficacy of prophylactic anticonvulsant drugs following traumatic brain injury: A systematic review and network meta-analysis of randomized controlled trials
Source: PLoS One. 2022 Mar 31;17(3):e0265932. doi: 10.1371/journal.pone.0265932 (PMC8970384; doi:10.1371/journal.pone.0265932)
Supplement: Attachment — Submitted filename: response to reviewers comment-0217 HYC.docx [file pone.0265932.s013.docx]

Reviewer #1: The authors should be congratulated for providing an excellent revision, which has substantially improved the rigour of this meta-analysis. My concerns have been largely addressed by this revision. This is an interesting paper that provides useful data regarding the comparative efficacy of various antiepileptic medications as seizure prophylaxis in traumatic brain injury.
Response: Thank you for your valuable comments that have significantly improved our manuscript. We have revised our manuscript based on your suggestions. The point-by-point responses to your specific comments are listed as below. We hope that our revisions are acceptable and that our responses adequately address the comments.

Major issues:
1. The authors have now added to the conclusion that they do not believe their findings support guidelines advocating the use of prophylactic phenytoin in traumatic brain injury. This is not supported by the data. Both the referenced Cochrane review, and the authors' own results, show that antiepileptics reduce the likelihood of early seizures. While there is currently no strong evidence to support any specific medication, phenytoin has the largest body of evidence for its efficacy and performed well in this meta-analysis despite the lack of statistical significance.

In addition, the efficacy of phenytoin relative to placebo IS arguably significant (OR 0.43, 95%CI 0.18-1.01). I would encourage the authors not to over-interpret p-values and the concept of 'statistical significance' in the context of network meta-analysis, where comparisons inevitably suffer from multiplicity (see doi: 10.1002/jrsm.1377). Therefore, the level IIA recommendation in the Brain Trauma Foundation guidelines to give one week seizure prophylaxis with phenytoin remains supported by the data in this study.

This is a good study but the conclusion in its current form is misleading and even potentially harmful because it implies that prophylaxis should not be given. The conclusion should be modified to make it clear that antiepileptic drugs reduce the risk of early seizures and the findings of this meta-analysis support current guidelines, but that the optimal medication is unclear. This can be considered a minor or major issue at the discretion of the editor, but should be rectified prior to publication.
Response: Thank you for the thoughtful comments. We agree with that the conclusion should be modified as that antiepileptic drugs reduce the risk of early seizures and the findings of this meta-analysis support current guidelines, but that the optimal medication is unclear. All changes were marked in red.

Minor issues:
1. In the text, (page 9), the effect of phenytoin relative to placebo in reducing early seizures is described as OR 0.43, 95%CI 0.18-0.99. In the corresponding table S4, this is listed as OR 0.43, 95%CI 0.18-1.01. Please check these values and ensure they are correct and matching.

Response: This was a typo error. We have revised the effect of phenytoin relative to placebo in reducing early seizure as OR 0.43, 95%CI 0.18-1.01, which corresponded to table S4 (page 9).

2. The results of the pairwise analysis comparing all AEDs versus placebo should also be included in the abstract, as the results of the paper cannot be properly interpreted without this knowledge. Response: We have added the results of the pairwise analysis comparing all AEDs versus placebo in the abstract. All changes were marked in red.
